# Gathering, agriculture, and exchange: an ethnoecological approach to the study of food patterns and feedstuff sources in communities of the Central Andes, Peru

**DOI:** 10.1186/s13002-024-00705-9

**Published:** 2024-07-24

**Authors:** Marggiori Pancorbo-Olivera, Fabiola Parra-Rondinel, Juan Torres-Guevara, Aldo Cruz-Soriano, Alejandro Casas

**Affiliations:** 1 Centro de Investigaciones en Zonas Áridas (CIZA), Universidad Nacional Agraria la Molina (UNALM), Lima, Peru; 2 Departamento Académico de Biología, Facultad de Ciencias, Universidad Nacional Agraria la Molina, Lima, Peru; 3 Coordinadora de Ciencia y Tecnología en los Andes, Lima, Peru; 4 Instituto de Investigaciones en Ecosistemas y Sustentabilidad, Universidad Nacional Autónoma de México, UNAM, Campus Morelia, Michoacán, Mexico

**Keywords:** Food patterns, Food sovereignty, Plant gathering, Peruvian Andean region, Risk management, Sustainable food systems, Wild, weedy, and ruderal edible plants

## Abstract

**Background:**

Historically, the Andean people have experienced uncertainty in terms of the availability of food resources because of climatic and ecological variations that are typical of mountainous environments. Risk management strategies, including the diversified and complementary use and management of species and ecosystems at different elevations, have faced such uncertainty. The current effects of climate change on food security motivate studies on subsistence adaptative strategies. TEK offers extraordinary experience and local biocultural memory to meet present and future needs. From an ethnoecological perspective, we aim to identify the variety of local foods in Andean communities, their cultural and nutritional value for local people, their use frequencies, and their forms to obtain them from different environments, productive systems, and interchanges. We expected to identify traditional Andean diversified subsistence patterns despite the pressure of modern food and interchange systems.

**Methods:**

This study was conducted in two communities in the highlands of the Department of Huánuco, Peru. We conducted 24 semistructured interviews with households sampled through the snowball method. We asked about their daily life food, plant and animal components of diet, frequencies and seasons in which they are consumed, and ways to obtain them. We complemented the information through ethnobotanical collection of wild, weedy, and ruderal edible plants and records on domestic and wild animals included in the diet.

**Results:**

We recorded 37 crop species, 13 domestic animals, 151 wild, weedy, and ruderal food plant species, the 3 most commonly consumed wild animals, and 52 processed products obtained from local stores and markets. The main crops are potato and maize, while the main domestic animals included in the diet are cattle, pigs, and sheep. Rice, pasta, and bread are the main raw and processed foods included in the diet. Crops represent nearly half of the food consumed and purchased (in kg/year), and tubers and cereals provide most of the kilocalories, carbohydrates and proteins. Wild, weedy, and ruderal plants are consumed in relatively low amounts and at relatively low frequencies per species, but overall, they constitute a significant proportion of the kg of annually consumed food (14.4% in Cani and 9.6% in Monte Azul). Knowledge and use of these resources play a key role in local cuisine and nutrition.

**Conclusion:**

The current food patterns studied are based on diverse diets, including multiple feedstuffs, sources, and practices to obtain them, which reflects the traditional Andean subsistence pattern. The increasing adoption of processed food has influenced the declining consumption of local food, mainly among young people. Communication and policies to promote local food, emphasizing the role of wild plants and their adequate consumption, and provide information on their nutritional value are recommended to support efforts toward food sovereignty and conservation of Andean biocultural diversity.

**Supplementary Information:**

The online version contains supplementary material available at 10.1186/s13002-024-00705-9.

## Background

The Andean region of Peru is currently inhabited by people whose cultures have approximately 10,000 years of agricultural tradition; among them the Chavín, Pucara, Huari, Chanca, Tiawanaku, and Wanka cultures for Peru can be mentioned [1, 2]. During this period, the Andean peoples domesticated potatoes (*Solanum* spp.), *oca* (*Oxalis tuberosa* Molina), *mashua* (*Tropaeolum tuberosum* Ruiz & Pav.), *olluco* (*Ullucus tuberosus* Caldas), *kiwicha* (*Amaranthus caudatus* L.), *tarwi* (*Lupinus mutabilis* Sweet), beans (*Phaseolus* spp.), *quinoa* (*Chenopodium quinoa* Willd.) among other important crops [3]. With more than millennia of domestication, each of these crops has accumulated high levels of morphological and genetic variation [4–6]. In addition, native animals such as *Lama glama* L., 1758, *Vicugna pacos* L., 1758, and *Cavia porcellus* L., 1758 were also domesticated in the region [7, 8]. Numerous other species of animals and plants have been gathered, hunted, managed, raised, and consumed by the Andean people since pre-Columbian times. Archeological research has been prolific, documenting species of organisms included in the human diet since prehistory [3]. Additionally, writings of the chroniclers of the conquest and colonial periods are informative about the broad spectrum of food consumed by Andean people just before European contact [9], while ethnobiological and health studies have described the use of a high number of native components of people’s current diet that were used in the past [10–13].

The organization of agricultural and silvicultural production, the distribution of products, and the sustainability of the Andean food system are based on the management of a high diversity of food products from multiple ecosystems [9, 14]. However, Spanish colonization caused numerous native food products to be, in some cases violently, replaced by Old World elements, many of which were inaccessible to indigenous people. This situation influenced changes in the diets of Andean settlers, who impoverished their food [9]. However, despite these historical facts, the culture of diversity and diversification continues to persist in the Andes. For instance, Brack [7] reported that in Peru, native peoples currently include nearly 5000 plant species, 787 of which are used as food (107 crops, 167 wild plants that are semicultivated, and the rest being wild species extracted from forests). Most of them (nearly 70%) are Amazonian plant species, but the remaining 30% include more than 400 species of Andean edible plants [7, 15]. This number will most likely increase with the progress of new studies, as we discuss below.

Historically, Andean agricultural systems have faced poor soils, geoecological diversity, and climatic uncertainty [16]. Consequently, for numerous authors, studying the traditional diversification of crops and agricultural techniques as strategies to address difficult environmental conditions and manage risk is the main issues of concern. Murra [17] described an Andean pattern of using resources and ecosystems, which he called the “vertical control of a maximum of ecological levels,” considering the variation in ecological conditions occurring at different elevations in the mountains [18]. Earls [16] called such a pattern the “massive parallelism strategy,” which refers to the spatial and temporal diversification of tasks. Current climate change involves high, increasing uncertainty [19], and peasants perceive new changes at the microclimatic level [20]. This fact allows for the supposition of expanding impacts on food security, which deserve to be analyzed from the perspective of processes of food system adaptation [21], including broadening the spectrum of assets [22].

Ethnoecological studies may significantly contribute to the recognition of forgotten or poorly known food resources, their sources and techniques for assessing them, and their uses, forms of preparation and management, nutritional value, availability, and potentialities to increase their adoption [23]. These studies need to be enhanced [24], especially those focused on wild edible plants [25–27], since these offer a broad spectrum of products. Studies related to traditional management are also important since local techniques are valuable for ensuring and increasing accessibility to those resources [28–30].

This study aimed to characterize local diet patterns in villages of the Central Andes of Perú; the role of wild, weedy, and cultivated food; the frequencies and amounts used; and the ways in which these diets are managed and obtained from local environmental units and markets. Based on previous studies on traditional subsistence strategies among mountain people in Peru [6, 17, 24, 31], we hypothesized the occurrence of a traditional diversified food pattern, based on a high variety of cultivated plants conforming to a basic diet, which is complemented by a broad spectrum of wild and weedy edible plants and animals obtained from gathering and hunting involving management strategies in different ecosystems.

## Methods

### Study area

**Fig. 1:**
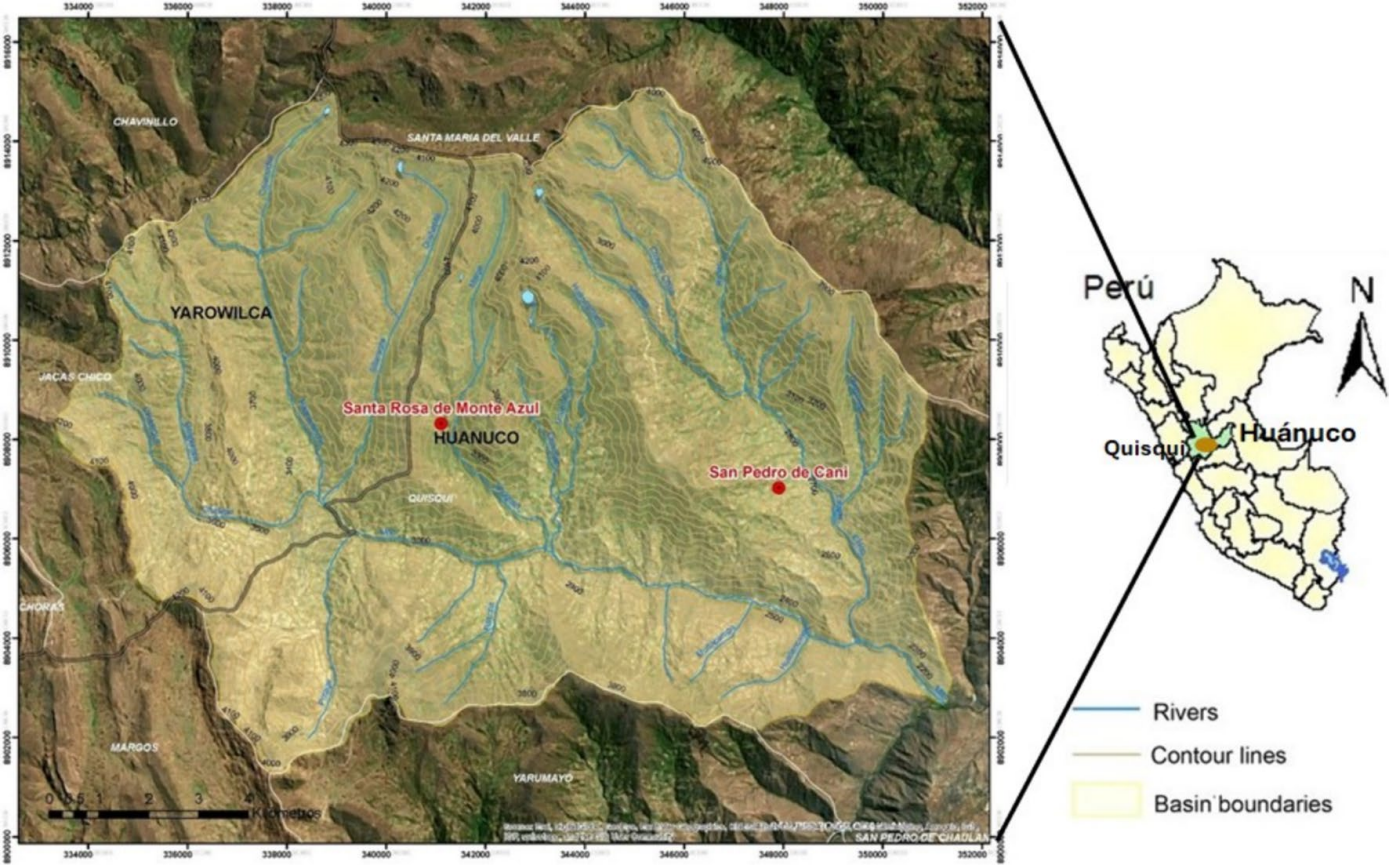
Location of the Mito watershed and the studied communities of Monte Azul and Cani in the Department of Huánuco, Perú. Scale: 1:60,000. Modified from Pancorbo-Olivera et al. [33]

This study was conducted in the Santa Rosa de Monte Azul (hereinafter Monte Azul) and San Pedro de Cani (hereinafter Cani) communities, both of which are located in the Mito watershed in Quisqui District, Department of Huánuco (Fig. 1). Mito is at the central Sierra of Peru, on the Andean eastern slope [32] at 09° 48′–09°55′ S and 76° 21′–76° 30′ W [6]. Both are Quechua communities, most of which are bilingual, speaking Quechua in their daily life and Spanish.

Monte Azul village is located at an elevation of approximately 3200 m, and its territory at lower and higher elevations in the Guellaymayo microbasin, characterized by the occurrence of a mountainous landscape with peaks, gorges, rivers, and slope topography [6]. The vegetation on the slopes is composed of brushwood and humid savannah, which are dominated by bushy species such as *Baccharis salicifolia* (Ruiz & Pavón) Pers., and relics of natural riparian forest dominated by *Alnus acuminata* Kunth and *Sambucus peruviana* Kunth, among other species. In addition, grass steppes, *puna* grasslands, and *puna* lawns are in high-elevation zones above 4000 m, where numerous *cochas* (lagoons), high Andean lakes, and wetlands are found. In that zone, the predominant crops are Andean tubers [6, 32].

The community of Cani is located at 2800 m of elevation in the low- and middle-elevation zones of the Lanjas microbasin, which is characterized by a dry, temperate, cold climate. Soils in the area have hydric limitations, and the characteristic steep hills contribute to decreasing their productivity [34]. The vegetation is mainly formed by secondary forests disturbed by slash and burn, dominated by *Eucalyptus globulus* Labill. and *Alnus acuminata*, brushwoods, humid savannah with shrubs of the genus *Berberis* spp., *Baccharis* spp. and *Lupinus* spp., as well as puna grasslands and puna lawns at the highest elevations, above 4000 m. The main crops are maize, fruit trees such as peach, and vegetables [32, 33].

In both communities, rainfall ranges from 100 to 500 mm in Quisqui district (33 years data; [34]) mostly occurring from October to March, whereas frosts occur between June and August [35]. Most families practice rainfed and irrigated agriculture and livestock raising. Both communities lack potable water systems and sewer drainage services. According to PMA-CENEPRED [36], the District of Quisqui has a “very high” vulnerability to food insecurity in the face of natural phenomena, for example frost, heavy rain, drought, landslides, and increase in pests (VIAFFNN by the acronym of this term used in Spanish by institutions).

We selected the study villages because they were involved in several studies they authorized. These communities had participated in previous studies on in situ management of agrobiodiversity since the 1990s, and our research team had built up personal trust. Interviews were conducted in Spanish, and in some cases, we had the assistance of Quechua-speaking colleagues.

### Interviews

To conduct this research, we obtained permission from the local authorities and people who were interviewed. We identified people (men and women) recognized to have vast knowledge about edible food plants (14 in Cani, 10 in Monte Azul). We contacted them following snowball sampling [37]. We conducted in-depth semistructured interviews with them [38, 39], looking for analyzing food patterns and sources considering households as units of analysis. These interviews included questions about crops they use to cultivate and animals they raise. Additionally, we included questions on the products that are obtained through interchange or purchased in stores and markets, the frequencies and amounts of products used as food, and the period of their availability throughout the year.

The quantities consumed of the main edible resources were measured to calculate the monthly and annual consumption of every food product in kg per household. To estimate the importance of animal products, we asked about the types and number of pieces consumed in a year, and we related this information to the average weight of each product (e.g., sacrificed animal, bled, flayed, eviscerated, without head or limbs); for guinea pigs and hens, the entire body weight was used. To estimate the frequency of consumption, we recorded the number of times every food product was consumed per month and year. We finally estimated the nutritional contribution of every food product per weight unit based on tables published by CENAN [40–44].

We recorded, collected, and herborized specimens of wild, weedy, and ruderal plants consumed as food. Voucher specimens were deposited in the MOL herbarium at the Universidad Nacional Agraria La Molina, Perú, under Pancorbo-Olivera, M. collection numbers (a preliminary report can be found in [33]). We conducted semistructured interviews to the 24 households mentioned above, ethnobotanical walks, and botanical collections to document their uses, management forms, habitats, and seasonal availability. The spelling of the Quechua names was verified with the Dictionary of Huánuco Quechua *Rimaycuna* [45]. The nutrient content of the plants was estimated according to several studies previously reporting information on some feedstuffs [46–51]. Management forms were categorized following the classification of Casas et al. [52–56]. The habitats from which the plants were collected were classified according to [32]. The classification of crop uses and other edible plants was based on the studies by Casas et al. [52–54, 57]. Information on the seasonal availability of plants and their products is based on the interviews. All foods were placed into a group according to CENAN [40–44].

### Food types

For organizing the information about food types, we considered the classification developed by Casas et al. [23] as follows: (a) Beverages, including infusions and cold drinks. (b) Vegetables: leaves, flowers, and fruits that are sometimes consumed as the main or complementary meal. (c) Fruits: these fruits include fresh fruits. (d) Desserts and condiments: consumed as flavorings in some dishes or in occasional desserts. (e) Miscellaneous: plants collected and consumed in the field.

## Results

### The basic diet

**Fig. 2:**
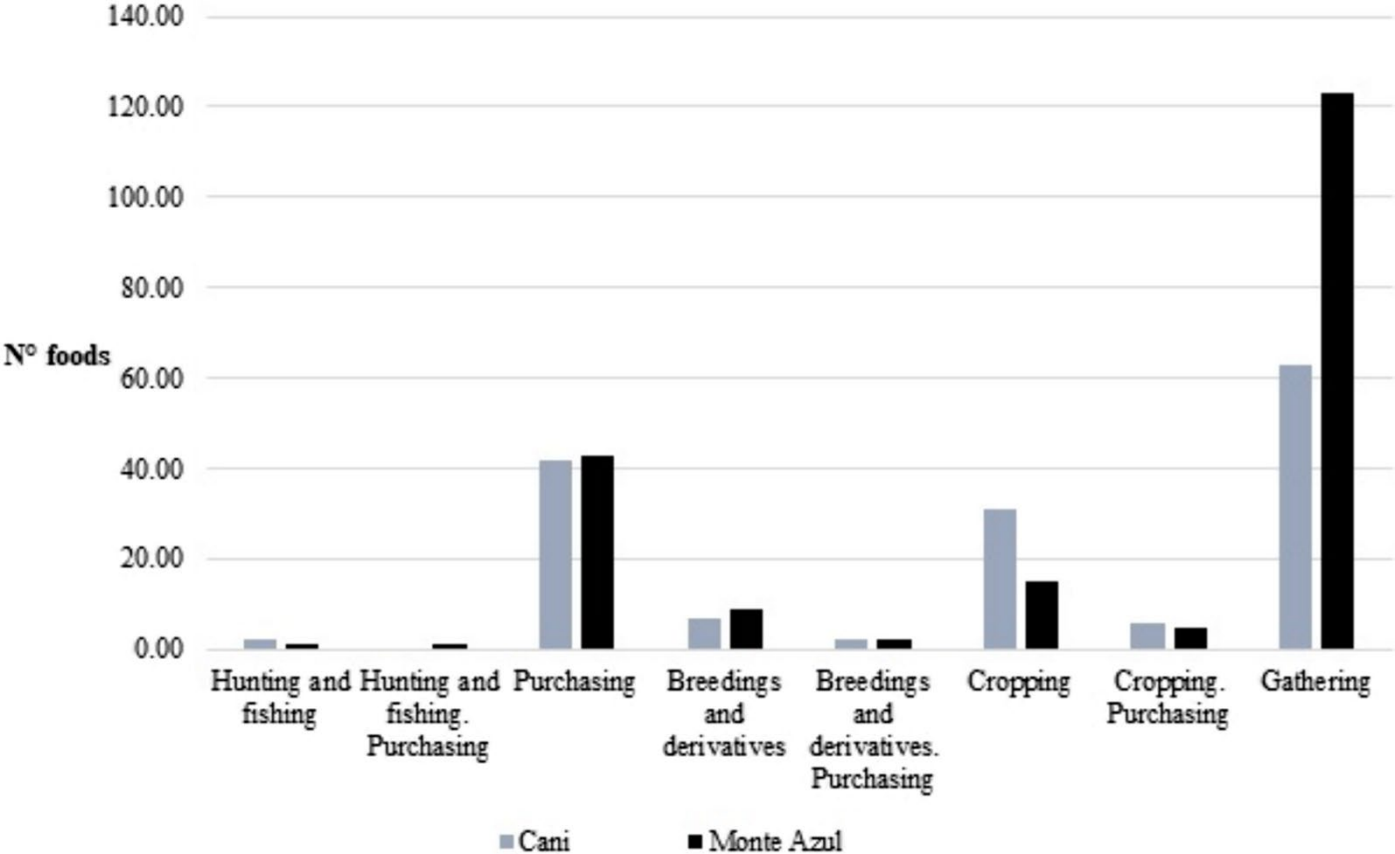
Number of food products according to their obtaining forms. The diversity of wild food products is much greater than that of crops and purchases, although the proportion of kilograms of food consumed per year is 14.4% in Cani and 9.6% in Monte Azul

We documented the consumption of a total of 133 types of food products in Cani and 189 in Monte Azul, all of which were obtained by cropping, animal raising, hunting, fishing, gathering, and purchasing feedstuffs in stores and markets. Several products are obtained through combinations of these activities. An important proportion of the consumed food products is exclusively purchased (31% in Cani, 24% in Monte Azul), and another considerable proportion is obtained from their own cropping activities (23% in Cani, 8% in Monte Azul). Nevertheless, people collect a high number of wild, weedy, and ruderal plants and provide a significant proportion of their diet. These plants are consumed in relatively low amounts and at relatively low frequencies per species, but overall, they constitute a significant proportion of the kg of annually consumed food (14.4% in Cani and 9.6% in Monte Azul). These plants are undoubtedly important components of the yearly diet and ingredients of local cuisine (Fig. 2). Even when there is a wide variety of food products available throughout the year in both communities, a basic, more frequent diet can be identified whose components are listed in Table 1. These are consumed by most households at relatively high frequencies and in relatively high amounts.

**Table 1:** Average weight of basic foods consumed during a day by a person in Cani and Monte Azul peasant communities

Source	San Pedro de Cani	Weight (g)	Santa Rosa de Monte Azul	Weight (g)
Crops and derivatives	Potatoes portion (parboiled, fried, in soups or stews)	800	Potatoes portion (parboiled, fried, in soups or stews)	760
	Corn portion *(cancha* or* mote)*	210	Plate of *Tocosh mazamorra*^a^	420
	Plate of *Tocosh mazamorra*^a^	350	Corn portion *(cancha* or* mote)*	120
			Portion of *Oca* (parboiled)	400
			Portion of *Olluco* (soups or stews)	1230
			Portion of *Mashua* (parboiled)	290
Products purchased in stores and markets	Rice portion (stews)	150	Rice portion (stews)	230
	Noodles portion (soups and stews)	150	Noodles portion (soups and stews)	210
	Bread portion	90	Brad portion	70
	Milk portion (cup of oatmeal or *mazamorra*)	90	Milk portion (cup of oatmeal, punch oatmeal, or *mazamorra*)	250
	Chicken portion (soups or stews)	300		
Domestic animals and derivatives	One egg	60	One egg	60
	Portion of hen (soups or stews)	300	Fresh cow’s milk (cup of mazamorra, punch oatmeal or cheese)	420

^a^Derivative product from some potato varieties (see Supplementary material 1)

### Food from crops

**Fig. 3:**
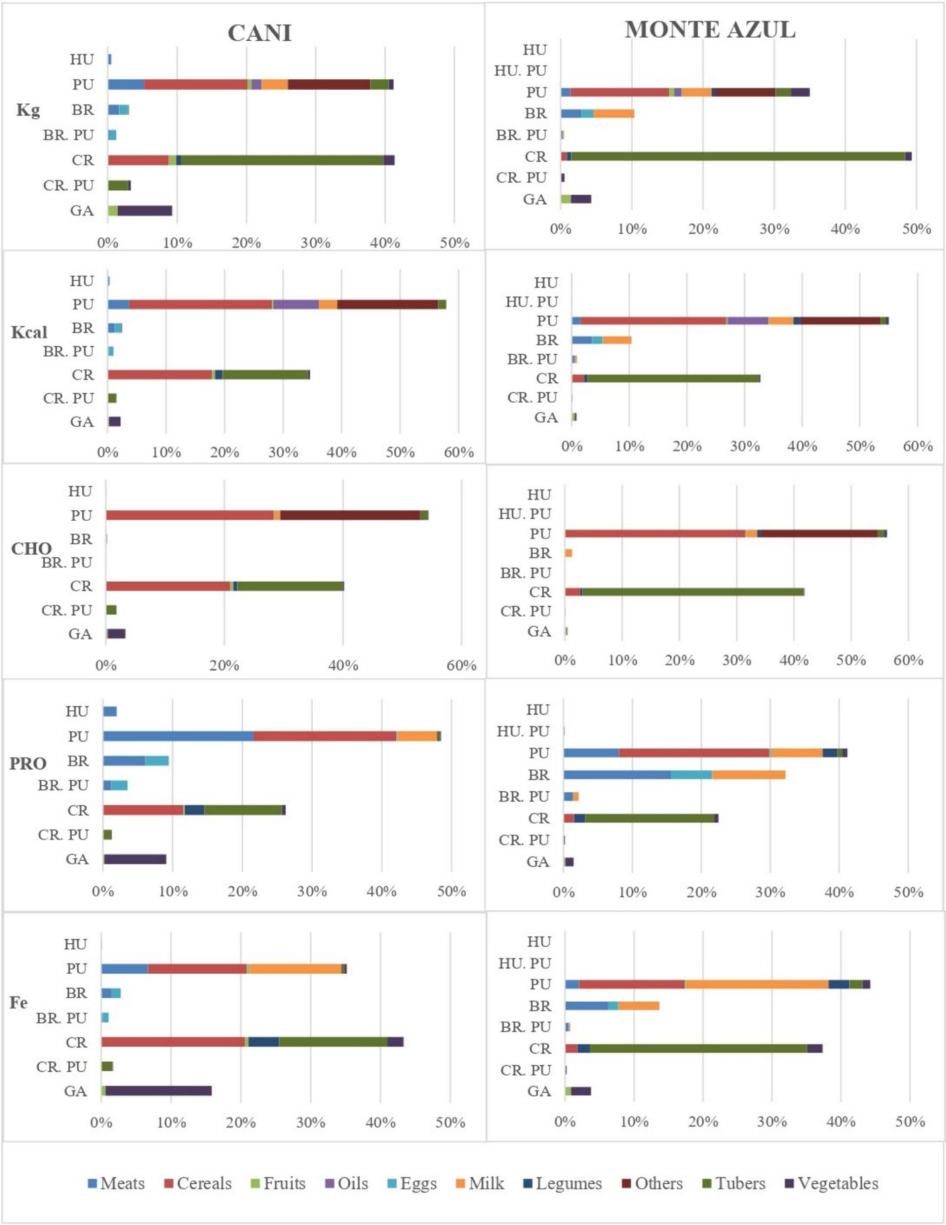
Percentages of kg, kcal, carbohydrates (g), proteins (g) and iron (mg) provided in one year by the documented foods according to their obtaining form and food group. The estimation of the contribution in kg, kcal, carbohydrates, proteins and iron obtained from gathered food was made based on 12 species, as indicated in Table 3, with the exception of berros (*Philoglossa* sp.), chulquillo (*Peperomia* sp.) and uchu uchu (*Ceratostema peruviana*), whose nutritional information could not be found. Nutritional contribution (%): Kg: Kilograms; Kcal: Kilocalories; CHO: Carbohydrates (g); PRO: Proteins (g); Fe: Iron (mg); Obtaining forms: HU, hunting and fishing; PU, purchasing; BR, breeding of domestic animals and derivatives; CR, crops and derivatives; GA, gathering

The main crops represent most of the weight of food (41.4% in kg) in people’s diet from Cani and 49.4% in Monte Azul. The agriculture and diet of Monte Azul are based on native varieties of potato, *oca*, *olluco*, and *mashua* crops, which are consumed in three main daily meals: parboiled, prepared as *tocosh* (Supplementary material 1); fried, in stews and soups (Supplementary material 2); and served with *cancha* (toasted corn) or *mote* (boiled threshed grains). The “improved” (modern) potato varieties, which are eventually consumed, are cropped in lowland areas (at elevations < 3000 m), but these varieties are mainly destined for sale. Crop tubers provide most of the total weight of food (47%), carbohydrates (38.6%), iron (31.51%), and proteins (18.8%) (Fig. 3). Other important crops are *Cucurbita ficifolia* Bouché, *Lupinus mutabilis* and *Chenopodium quinoa* (Table 2). In total, 44.4% of the vegetable crops grow in homegardens, and the most common are cabbage (*Brassica oleracea* var. *capitata*) and parsley (*Petroselinum crispum* (Mill.) Fuss). Legumes and vegetables are important sources of protein and iron, respectively.

**Table 2:** Cropped foods in Cani (CA) and Monte Azul (MO) communities

Local names	Family	Species	Use	Habitat	% Families		Harvest months											
					CA	MO	J	F	M	A	M	J	J	A	S	O	N	D
Cedron	Verbenaceae	*Aloysia citriodora* Paláu	B	H. F	7.1	0	X	X	X	X	X	X	X	X	X	X	X	X
Chamomile	Asteraceae	*Matricaria chamomilla* L	B	H	14.2	0	X	X	X	X	X	X	X	X	X	X	X	X
Balm	Lamiaceae	*Melissa officinalis* L	B	H. F	7.1	0	X	X	X	X	X	X	X	X	X	X	X	X
Culantro	Apiaceae	*Eryngium foetidum* L	D	H	14.3	20	X	X	X	X	X	X	X	X	X	X	X	X
Oregano	Lamiaceae	*Origanum vulgare* L	D	H	14.3	0	X	X	X	X	X	X	X	X	X	X	X	X
Ruta	Rutaceae	*Ruta graveolens* L	D	H	14.3	0	X	X	X	X	X	X	X	X	X	X	X	X
Custard apple	Annonaceae	*Annona cherimola* Mill	F	H	7.1	0					X	X						
Peach	Rosaceae	*Prunus persica (L.) Batsch*	F	H	14.3	0		X	X							X	X	
Passion fruit	Passifloraceae	*Passiflora ligularis* Juss	F	H	14.3	0		X	X									
Mandarin orange	Rutaceae	*Citrus reticulata* Blanco	F	H	7.1	0												X
Apple	Rosaceae	*Pyrus malus* L	F	H	7.1	0	X				X	X						X
Pea	Fabaceae	*Pisum sativum* L	L	H	28.6	0					X	X	X	X				
Chocho	Fabaceae	*Lupinus mutabilis* Sweet	L	H	7.1	20						X	X					
Bean	Fabaceae	*Phaseolus vulgaris* L	L	H	21.4	0						X	X	X	X			
Chickpea	Fabaceae	*Cicer arietinum* L	L	H	7.1	0				X	X							
Broad bean	Fabaceae	*Vicia faba* L	L	H	14.3	10		X	X									
Corn	Poaceae	*Zea mays* L	G	H	100	40					X	X	X	X				
Quinoa	Amaranthaceae	*Chenopodium quinoa* Willd	G	H	21.4	20						X	X	X				
Wheat	Poaceae	*Triticum spp.* L	G	H	14.3	0						X	X					
Arracacha	Apiaceae	*Arracacia xanthorrhiza* Bancr	R	H	7.1	0			X	X						X	X	
Beetroot	Amaranthaceae	*Beta vulgaris* L. ^a^	R	H. F	7.1	10												
Sweet potato	Convolvulaceae	*Ipomoea batatas* (L.) Lam,	R	H. F	7.1	0		X	X									
Mashua	Tropaeolaceae	*Tropaeolum tuberosum* Ruiz & Pav	R	F	7.1	60					X	X						
Oca	Oxalidaceae	*Oxalis tuberosa* Molina	R	F	21.4	70					X	X						
Olluco	Basellaceae	*Ullucus tuberosus* Caldas	R	F	21.4	70					X	X						
Potato	Solanaceae	*Solanum spp.* L	R	F	100	100				X	X							
Radish	Brassicaceae	*Raphanus sativus* L	R	H. F	7.1	0	X	X	X	X	X	X	X	X	X	X	X	X
Tocosh potato	Solanaceae	*Solanum spp.* L	R	F	21.4	90	X	X	X	X	X	X	X	X	X	X	X	X
Caigua	Cucurbitaceae	*Cyclanthera pedata* (l.) Schrad	V	F	21.4	0	X	X						X	X			
Pumpkin	Cucurbitaceae	*Cucurbita ficifolia* Bouché	V	F	64.3	30		X										
Welsh onion	Amaryllidaceae	*Allium fistulosum* (L.)	V	H. F	14.3	10	X	X	X	X	X	X	X	X	X	X	X	X
Cabbage	Brassicaceae	*Brassica oleracea var. capitata* L. ^a^	V	H	21.4	10												
Colish	Brassicaceae	*Brassica sp.* L	V	H	0	30	X	X	X	X	X	X	X	X	X	X	X	X
Lettuce	Asteraceae	*Lactuca sativa* L	V	H. F	0	10	X	X	X	X	X	X	X	X	X	X	X	X
Avocado	Lauraceae	*Persea americana* Mill	V	F	14.3	0	X	X	X									X
Parsley	Apiaceae	*Petroselinum crispum* (Mill.) Fuss	V	H	0	20	X	X	X	X	X	X	X	X	X	X	X	X
Carrot	Apiaceae	*Daucus carota var. sativus* Hoffm.^a^	V	H. F	7.1	10												
Squash	Cucurbitaceae	*Cucurbita maxima* Duchesne^b^	V	F	14.3	0												

Numbers in parentheses are Pancorbo-Olivera collection numbers [33] deposited in the herbarium MOL at the Universidad Nacional Agraria La Molina, Lima, Perú

^a^Its harvest depends on the sowing time. It is available five months per year

^b^It is sowed twice a year, at any month. Each harvest last around two months

Use: F, fruits; V, vegetables; B, beverages; D, desserts and condiments; L, legumes; G, grains or cereals; R, roots, bulbs, and tubers

Habitat: H, homegarden; F, farm

**Fig. 4:**
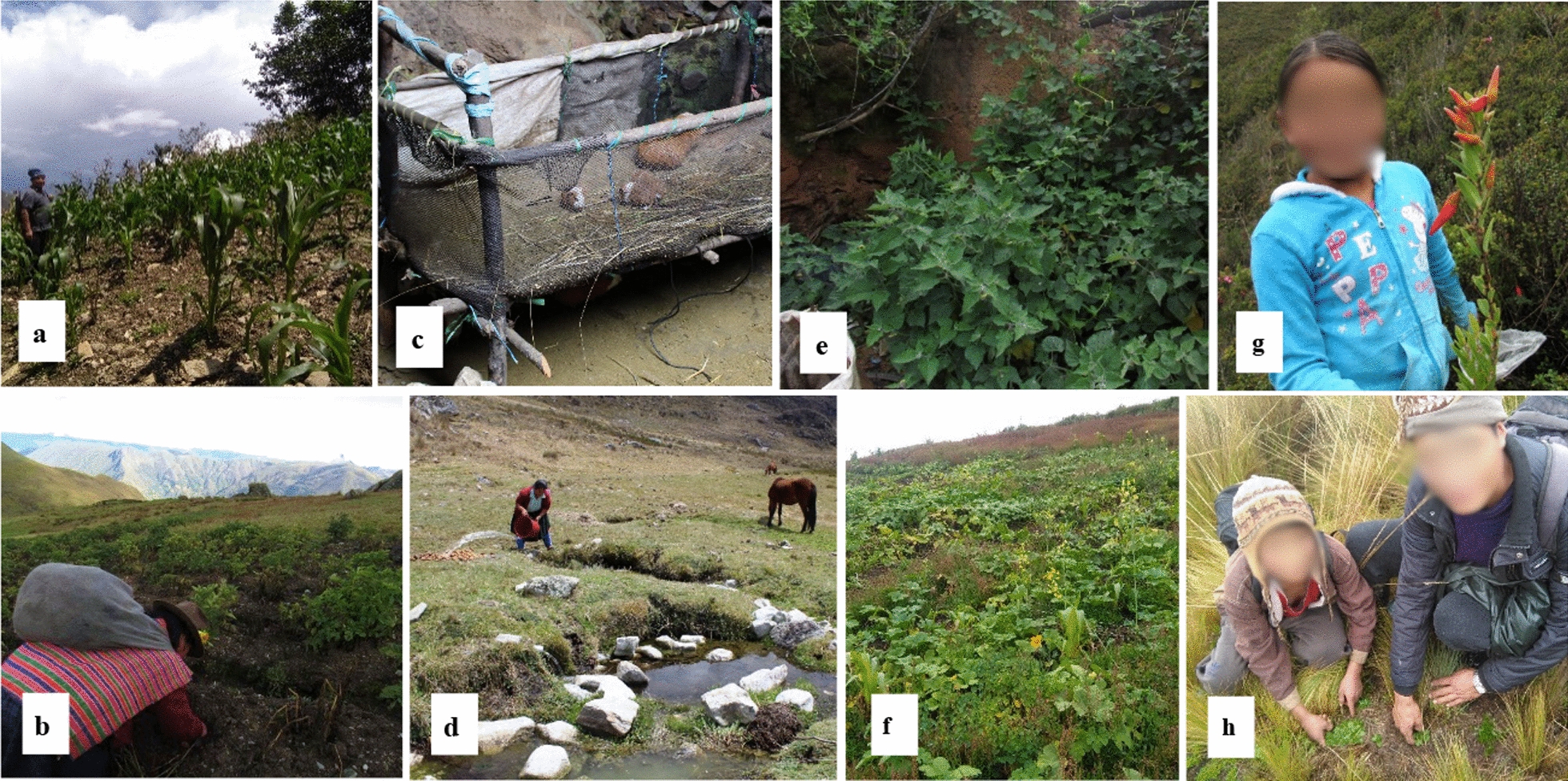
Activities related to food obtaining in Cani and Monte Azul. **a** Corn cropping in Cani. **b** Potato cropping in a *huachuy* system (variety mixture) in Monte Azul. **c** Guinea pig (*Cavia porcellus*) raised in Monte Azul. **d** *Tocosh* wells in Monte Azul. **e** Q*uishiu* (*Cyclanthera brachybotrys*) and *capulí* (*Physalis peruviana*) in a homegarden. **f** *Yuyo* (*Brassica rapa* subsp. *campestris*) on a crop and pumpkin farm. **g** Collection of *uchu uchu* (*Ceratostema peruviana*) in Monte Azul. **h** Collection of *walmish* (*Senecio condimentarium*) in the high zones of Cani. Photos: Marggiori Pancorbo-Olivera

Agriculture in Cani is based on maize, which is sometimes associated with *C. ficifolia* and beans (*Phaseolus vulgaris* L.). Corn is consumed as *cancha*, *mote*, *chicha morada* (a beverage made of a purple variety of maize), or *tocosh*, which is fermented maize prepared similarly to potato *tocosh * (See Fig. 4a, b and d for maize field, potato field and potato *tocosh *wells, respectively). Maize, wheat, and quinoa provide a large proportion of kcal (17.9%), carbohydrates (21%), proteins (11.6%), and iron (20.6%). Pumpkins are mainly consumed as *mazamorra* (sweet and thick dessert, such as porridge) and several stews, while beans are only consumed boiled and cooked as stews (Supplementary material 2). Modern potato varieties are the most commonly grown and consumed in this village. Potato, *oca, mashua* and *olluco*, provided 29.1% of the consumed weight, 14.6% of kcal, 17.7% of carbohydrates, 11.2% of proteins, and 15.5% of iron (Fig. 3). This community is also a producer of broad beans (*Vicia faba* L.), peas (*Pisum sativum* L.), and fruits such as peach (*Prunus persica* (L.) Stokes.), apple (*Malus domestica* Borkh.), and passion fruit (*Passiflora ligularis* Juss.). Among the crops, 28.6% are greens, which are mainly cultivated in home gardens (61.5% of the crops in this system); the most common are culantro (*Coriandrum sativum* L.) and oregano (*Origanum vulgare* L.), or in special spaces inside the chakras, the Welsh onion (*Allium fistulosum* L.) and carrots (*Daucus carota* L.). Selling crops generate monetary income for people, who are accustomed to buying complementary foods (Table 2).

### Food from domestic animals

Domestic animals provide 3% and 10% of the kilograms of food consumed per year in Cani and Monte Azul, respectively, and 2 and 8% of the kcal, respectively (Fig. 3). Most families in Monte Azul own cattle, sheep, pigs, hens, and guinea pigs (*Cavia porcellus*, Fig. 4c). They use soups and stews with eggs, milk, and fresh cheese. Sheep, pig, and beef meats are prepared as *charqui* (dry meat) and are occasionally consumed. Meat, milk, and eggs provide 15.6, 10.62, and 6% of the total protein, respectively, which means nearly one-third of the total protein consumed annually.

These animals are raised in Cani but in lower numbers. Only hens are numerous in the backyards of most houses; therefore, eggs (3.3% of the proteins) and hen meat (5%) are consumed more frequently than in Monte Azul. In both communities, the main consumption of fresh meat occurs during festivities, when it is cooked as *pachamanca* or *locr*o (Supplementary material 2).

### Food from stores and markets

In Monte Azul, stores and the market provide 31% of the annual weight of food consumed, 42% of kcal and 44.3% of iron. Purchased cereals or their products (rice, noodles, bread, corn, wheat) stand out since they provide 1/4 of kcal, 1/3 of carbohydrates, 21.8% of the proteins, and 15.4% of iron annually consumed. Fish, lamb, beef, and chicken meat (8.1% of proteins) and milk (7.4%), as well as a variety of vegetables, greens and fruits, with which people prepare their dishes, are also purchased; squashes, mandarin, and tomatoes are among the most common (Fig. 3). In Cani, 41.2% of the food weight consumed per year is purchased and provides 58.9% of the kcal. Purchased cereals (rice, noodles, bread, cookies) provide high amounts of kcal (24.5%), carbohydrates (28.3%), proteins (20.5%), and iron (14.16%). Although local people own cattle, most milk and cheese (5.56% of proteins) consumed are bought; similarly, approximately 14.1% of chicken meat is obtained on the market. Other products, such as banana, orange, tomato and lemon, and vegetables, are also purchased (Fig. 3).

Salt and sugar are in the category “others.” Salt (no calories) and oil (7% of kcal in both communities) are used daily in soups, stews, *cancha* and fries, whereas sugar (13.9% of kcal in Monte Azul, 17.4% in Cani) is used in infusions and desserts (Fig. 3). Occasionally, lunch may be only parboiled potatoes served with salt or *ají* (Supplementary material 2).

The households interviewed said to purchase groceries in the market of Huánuco (the capital city of the Department) and in grocery stores of Huancapallac (the capital city of the District). In Cani, there are grocery stores where *tocosh, oca, olluco* and *mashua* can be purchased. The owners of the stores, in turn, buy these products to people who come from highland villages.

### Hunting and fishing

These activities are not significant for the local people’s diet. Deer hunting was reported to occur occasionally only in Cani, and *vizcacha* (*Lagidium viscacia* Molina, 1782; Chinchillidae) hunting was observed in Monte Azul. Trout (*Orcorhynchus* spp.) fishing is forbidden in Monte Azul since settlers have perceived a decrease in its population in recent years. However, trout are occasionally fished for celebrating special events.

### Wild, weedy, and ruderal food plants

Together, wild, weedy, and ruderal plants provide an important proportion of the total annual amount of food in kg, 14.4% in Cani, and 9.6% in Monte Azul. In this study, we identified a total of 149 edible species of wild, weedy, and ruderal plants belonging to 47 botanical families, the most representative of which were Asteraceae (23.3%), Lamiaceae (7.33%), Ericaceae (6%), and Rubiaceae (4.7%).

*Use forms.* Some wild, weedy, and ruderal plants are considered part of the basic food. Twelve species are frequently gathered in relatively large quantities to prepare different dishes (Supplementary material 2); they provide 9 and 4% of the total weight of food (in kg) (2% and 1% of kcal, respectively) annually consumed in Cani and Monte Azul, respectively (Fig. 3). As shown in Table 3, five weedy and 5 ruderal plant species were identified, which indicates that crop fields and surrounding areas are important places for obtaining noncrop food plants.

**Table 3:** Main wild, weedy, and ruderal plants consumed in Cani and Monte Azul communities

Food	Local names	Family	Species	Use	Habitat	E. S	Management	Collecting months											
								J	F	M	A	M	J	J	A	S	O	N	D
B	Atogo	Amaranthaceae	*Amaranthus hybridus* L. (5)	V	F. H. S	We	C. To. E					X	X	X	X				
A	Berros	Brassicaceae	*Nasturtium officinale* W. T. Aiton (30)	V. B	F	Wi	C. Tr	X	X	X	X	X	X	X	X	X	X	X	X
S	Chincho, purun chincho	Asteraceae	*Tagetes elliptica* Sm. (39)	V. D	S. H	Wi. R	C. To. Tr	X	X	X	X	X	X	X	X	X	X	X	X
I	Uchu uchu	Ericaceae	*Ceratostema* aff. *peruviana* (59)	B. F. D	G	Wi	C	X	X	X									
C	Puka satu	Ericaceae	*Thibaudia mellifera* Ruiz & Pav. ex J. St.-Hil. (60)	F	G	Wi	C		X	X									
	Gongapa	Ericaceae	*Vaccinium floribundum* Kunth (61)	F	G	Wi	C	X	X	X									X
F	Yuyo, jitcka	Brassicaceae	*Brassica rapa* L. subsp. *campestris* (44)	V. B	F. S	Wi. We	To. E		X	X	X	X	X						
O	Quishiú	Cucurbitaceae	*Cyclanthera brachybotrys* (Poepp. & Endl.) Cogn. (53)	V. D	F. H	We	E. To. S		X	X									
O	Ucush-puru, Jirka-puru puru	Passifloraceae	*Passiflora tripartita* var. *mollisima* (Kunth) Holm-Niels. & P. Jørg. (103)	F	F. H. Ri	Wi. R	C		X	X	X								
D	Chulquilla (o), Ulluchana	Piperaceae	*Peperomia crystallina* Ruiz & Pav. (106)	V. D	F. Ri	Wi. R	C	X	X	X	X	X							
	Mulaca, shiraca, Shira mullaca	Rosaceae	*Rubus coriaceus* Por (126)	F. B. D	F. Ro. S. Ri	Wi. R. We	C. To		X	X	X	X							
	Mulaca, shiraca, Shira mullaca	Rosaceae	*Rubus floribundus* Weihe (127)	F. B. D	Ro. F	Wi. R. We	C. To		X	X	X	X							
C	Rallán, sauco	Adoxaceae	*Sambucus peruviana* Kunth (1)	F. D	H. F	Wi. R	C. Tr. S	X	X	X								X	X
O	Papayita de monte	Alstroemeriaceae	*Bomarea brevis* (Herb.) Baker (2)	F	Ri	Wi	C					X	X	X	X				
M	Pachag pachag	Alstroemeriaceae	*Bomarea cornigera* Herb. (3)	B	Ri	Wi	C	X	X	X	X	X	X	X	X	X	X	X	X
P	Cashuá, paico, camatay	Amaranthaceae	*Dysphania ambrosioides* (L.) Mosyakin & Clemants (6)	V. B	F. S	Wi. We	C. To. S	X	X	X	X	X				X	X	X	X
L	Jirka comino. Pacha perejil	Apiaceae	*Eryngium humile* Cav. (8)	B. D	F	We	To	X	X	X						X	X	X	X
E	Ratamsha	Rosaceae	*Acaena ovalifolia* Ruiz & Pav. (119)	B	S	Wi	C	X	X	X	X	X	X	X	X	X	X	X	X
M	Jacha arnika macho	Asteraceae	*Achyrocline alata* (Kunth) DC. (11)	B	S	Wi	C	X	X	X	X	X	X	X	X	X	X	X	X
E	Pacha arnika	Asteraceae	*Achyrocline* sp. 1	B	G	Wi	C	X	X	X	X	X	X	X	X	X	X	X	X
N	Jacha arnika hembra	Asteraceae	*Achyrocline* sp. 2	B	G	Wi	C	X	X	X	X	X	X	X	X	X	X	X	X
T	Jirka walmish, walmish walmish	Asteraceae	*Ageratina sternbergiana* (DC.) R.M. King & h.Rob (12)	B	Ro	R	C. To	X	X	X	X	X	X	X	X	X	X	X	X
A	Taya hembra, Taya macho	Asteraceae	*Baccharis chilco* Kunth (14)	B	S	Wi	C	X	X	X	X	X	X	X	X	X	X	X	X
R	Hierba de venado, San José	Asteraceae	*Baccharis buxifolia* (Lam.) Pers. (13)	B	F	We	C. To	X	X	X	X	X	X	X	X	X	X	X	X
Y	Tres esquinas, ututu, cuchu cuchu	Asteraceae	*Bacharis genistelloides* (Lam,) Pers. (15)	B	S	Wi	C	X	X	X								X	X
	Shillcu blanco, shillcu hembra	Asteraceae	*Bidens andicola* Kunth (17)	B	Ri	Wi	C					X	X	X	X				
F	Shillcu rojo, shillcu macho	Asteraceae	*Bidens pilosa* L (18)	B	Ri	Wi	C					X	X	X	X				
O	Shillcu amarillo, shillcu macho	Asteraceae	*Bidens* sp. (19)	B	F. Ri	Wi. We	C. To	X	X	X								X	X
O	Huamanpinka	Asteraceae	*Chuquiraga raimondiana* A. Granda (21)	B	G	Wi	C			X	X	X							
D	Chicoria	Asteraceae	*Taraxacum officinale* F.H. Wigg. (41)	B	Ro	R	C	X	X								X	X	X
	Matara	Asteraceae	*Loricaria thuyides* (Lam.) Sch. Bip. (27)	B	L	Wi	C					X	X	X	X				
	Campogana, yampogana	Asteraceae	*Sonchus oleraceus* L. (35)	V. B	F	We	C	X	X	X							X	X	X
	Cardón cardón	Asteraceae	*Senecio canescens* (Bonpl.) Cuatrec (33)	B	S. Ro	Wi. R	C	X	X	X	X	X	X	X	X	X	X	X	X
	Sacha canela, jacha canela	Rosaceae	*Geum peruvianum* Focke (121)	B	F	We. R	C. To	X	X	X	X	X	X	X	X	X	X	X	X
	Yuraj jachi/Gangush	Asteraceae	NN 5	B. M	S	Wi	C	X	X	X						X	X	X	X
	Palito de goma	Malvaceae	*Triumfetta calycina* Turcz (86)	B	S. F	Wi	C	X	X	X	X	X	X	X	X	X	X	X	X
	Yampogana	Asteraceae	*Sonchus oleraceus* (35)	V. B	G	Wi	To	X	X	X	X	X	X	X	X	X	X	X	X
	Lucho pringrin	Asteraceae	*Hieracium* sp. (25)	B	G	Wi	C	X	X	X	X	X	X	X	X	X	X	X	X
	Potga, jirka potga	Asteraceae	*Senecio burkartii* Cabrera (31)	B	G	Wi	C	X	X	X	X	X	X	X	X	X	X	X	X
	Frescosnero, descorsonero	Asteraceae	*Perezia multiflora* (Bonpl.) Less. (29)	B	G	Wi	C. To	X	X	X							X	X	X
	Huiro huiro	Asteraceae	*Senecio canescens* (Bonpl.) Cuatrec. (33)	B	G	Wi	C	X	X	X	X	X	X	X	X	X	X	X	X
	Lancausha (u, o), llancausha (u, o)	Asteraceae	*Senecio rhizomatus* Rusby (32)	B	G	Wi	C	X	X	X	X	X	X	X	X	X	X	X	X
	Lancausha (u, o), llancausha (u, o)	Asteraceae	*Senecio* aff. *robustus* (36)	B	G	Wi	C	X	X	X	X	X	X	X	X	X	X	X	X
	Walmish, anomakey	Asteraceae	*Senecio condimentarius* Cabrera (34)	V. D	G	Wi	C	X	X	X	X	X	X	X	X	X	X	X	X
	Chinaca huiro huiro	Asteraceae	*Chrysactinium amphothrix* (S.F	B	S	Wi	C	X	X	X	X	X	X	X	X	X	X	X	X
			Blake) H. Rob. &																
			Brettell (20)																
	Chinaca huiro huiro	Asteraceae	*Senecio burkartii* Cabrera (31)	B	S	Wi	C	X	X	X	X	X	X	X	X	X	X	X	X
	Anís, anís de chacra, anís de monte	Asteraceae	*Tagetes filifolia* Tag. (40)	B. D	F. H	We	C. To. E. S		X	X									
	Chincho, shalla chincho	Asteraceae	*Tagetes minuta* L. (38)	D	S. H	Wi. We	To. Tr	X	X	X	X	X	X	X	X	X	X	X	X
	Shalla chincho	Asteraceae	*Tagetes elliptica* Sm. (39)	V. D	S. H	Wi. We	C. To. Tr	X	X	X	X	X	X	X	X	X	X	X	X
	Lutu ullucu, jupay olluco	Basellaceae	*Ullucus tuberosus* Caldas (42)	B	F	We	To	X	X	X									X
	Jacha colish blanco y amarillo	Brassicaceae	*Brassica napus* L. (43)	V	F	We	C. To. E				X	X	X	X					
	Bolsa bolsa	Brassicaceae	*Capsella bursa-pastoris* (L.) Medik. (45)	V	F	Wi	To				X	X	X	X	X				
	Pachan colish, shilma	Montiaceae	*Calandrinia ciliata* (Ruiz & Pav.) DC. (89)	V. D	F	We	To				X	X	X	X	X				
	Romero. Ucush-romero	Calceolariaceae	*Calceolaria linearis* Ruiz & Pav. (48)	B	S	Wi	C	X	X	X	X	X	X	X	X	X	X	X	X
	Yana ogoro	Calceolariaceae	*Calceolaria tenuis* Benth. (49)	B	Ri	Wi	C	X	X	X	X	X	X	X	X	X	X	X	X
	Ucush-papaya	Caricaceae	*Carica microcarpa* Jacq. (52)	F. D	F. H	Wi	C. To. E. S	X	X										
	Cola de caballo, mogo mogo	Equisetaceae	*Equisetum bogotense* Kunth (54)	B	F. Ri	Wi. We	C. To		X										
	Purun rosa	Ericaceae	*Bejaria aestuans* Mutis ex L. (55)	B	G	Wi	C	X	X	X	X	X	X	X	X	X	X	X	X
	Manayupa hembra, macho	Fabaceae	*Desmodium adscendens* (Sw.) DC. (64)	B	S	Wi	C		X	X									
	Manayupa macho	Fabaceae	*Desmodium molliculum* (Kunth) DC. (63)	B	S	Wi	C. To		X	X									
	Chocho, tarwi silvestre	Fabaceae	*Lupinus piurensis* C.P. Sm. (65)	V	S	Wi	C		X	X	X	X							
	Gallo, gallu gallu, rima rima	Fabaceae	*Dalea exilis* DC. (62)	B	F	R. We	C	X	X	X	X	X	X	X	X	X	X	X	X
	Culín hembra, culín, walwa	Fabaceae	*Otholobium glandulosum* (L.) J.W. Grimes (66)	B	F	We	C. E	X	X	X	X	X	X	X	X	X	X	X	X
	Chinchimalí, chinchimalí macho	Linaceae	*Linum polygaloides* Planch. (82)	B	Ro	R	C	X	X	X	X	X	X	X	X	X	X	X	X
	Pacha apio	Apiaceae	*Niphogeton azorelloides* Mathias &	B	L	Wi	C	X	X	X	X	X	X	X	X	X	X	X	X
			Constance (9)																
	Auja auja	Geraniaceae	*Erodium moschatum* (L.) L’Hér. (67)	B	F	We	C. E	X	X	X								X	X
	Auja auja	Geraniaceae	*Geranium* aff. *peuvianum*, Hieron. (68)	B	F	We	C. E	X	X	X								X	X
	Jirka pimienta, pimienta silvestre	Apiaceae	*Oreomyrrhis andicola* (Kunth) Endl. ex Hook.f. (10)	D	G	Wi	C	X	X							X	X	X	X
	Pichiuquita, chulmish	Lamiaceae	*Clinopodium breviflorum* (Benth.) Govaerts (70)	B	F	We	C	X	X	X	X	X	X	X	X	X	X	X	X
	Pichiuquita hembra, Psiguchaqui	Lamiaceae	*Clinopodium striatum* (Ruiz & Pav.) Govaerts (72)	B	F	We	C	X	X	X	X	X	X	X	X	X	X	X	X
	Pachasalvia	Lamiaceae	*Lepechinia meyenii* (Walp.) Epling (73)	B	F	We	C	X	X	X									X
	Herba buena olor	Lamiaceae	*Mentha spicata* L. (74)	V. B. D	S	Wi	C. Tr	X	X	X	X	X	X	X	X	X	X	X	X
	Muñá, yana muñá, muñá negra	Lamiaceae	*Minthostachys mollis* (Griseb. (76)	V. B	S. G. F. Ro	Wi. R. We	C. To	X	X	X	X	X	X	X	X	X	X	X	X
	Pacha muñá	Lamiaceae	*Clinopodium nubigenum* (Kunth) Kuntze (71))	B	G	Wi	C. To	X	X	X	X	X				X	X	X	X
	Yawar jutumi	Amaranthaceae	*Alternanthera porrigens* (Jacq.) Kuntze (4)	B	S. Ro	Wi. R	C	X	X										X
	Pacha muñá pichiuquita	Lamiaceae	*Clinopodium breviflorum* (Benth.) Govaerts (70),	V. B	S. G	S	C. To	X	X	X						X	X	X	X
	Salvia	Lamiaceae	*Salvia sagittata* Ruiz & Pav. (77)	B	F	We	C	X	X										X
	Papagora macho, Papagora hembra	Lamiaceae	*Stachys* aff. *pusilla* (Wedd.) Briq. (80)	B	F	A	C	X	X	X	X	X	X	X	X	X	X	X	X
	Papagora macho	Lamiaceae	*Stachys* aff. *herrerae* Epling (79)	B	G. S. F	Wi. A	C. To	X	X	X	X	X	X	X	X	X	X	X	X
	Pacha arnika, Chinchimalí macho	Asteraceae	*Achyrocline* aff. *alata* (Kunth) DC. (11)	B	S	Wi	C	X	X	X							X	X	X
	Aurej ishanca	Loasaceae	*Caiophora* aff. *cirsiifolia* K.Presl (83)	B	F. S	We. Wi	To	X	X	X	X	X	X	X	X	X	X	X	X
	Ishanca	Loasaceae	*Loasa* sp. 1	B	F. S	Wi. We	C	X	X	X	X	X	X	X	X	X	X	X	X
	Mula ishanca	Loasaceae	*Nasa grandiflora* (Desr.) Weigend (84)	B	F	We	C	X	X	X	X	X	X	X	X	X	X	X	X
	Borragas ishanca, campanilla ishanca	Loasaceae	*Nasa* sp.	B	F	We	C	X	X	X	X	X	X	X	X	X	X	X	X
	Antarragá	Montiaceae	*Calandrinia acaulis* Kunth (89)	B	F	We	C	X	X	X									
	Shullun orégano, tienda muñá, china orégano	Lamiaceae	*Minthostachys* aff. *spicata* (Benth.) Epling (77)	V. B	H	Wi	C. Tr	X	X	X	X	X	X	X	X	X	X	X	X
	Ñuñupuku	Campanulaceae	*Wahlenbergia* aff	B. M	G	Wi	C	X	X	X								X	X
			*urcosensis* E. Wimm. (51)																
	Shupla. Shuplanco (a)	Solanaceae	*Salpichroa* aff. *weberbauerii* Dammer (134)	F. B	L	Wi	C					X	X	X					
	Ichiqolgoy “donde, donde”	Onagraceae	*Fuchsia denticulata* Ruiz & Pav (92)	B	S	Wi	C	X	X	X	X	X	X	X	X	X	X	X	X
	Chupa sangre, Auja auja	Onagraceae	*Epilobium denticulatum* Ruiz & Pav. (91)	B	G. S. F	Wi	C. To	X	X	X									
	Chupa sangre, antañahui	Onagraceae	*Oenothera rosea* L’Hér. ex Aiton (94)	B	F. L. S	Wi. R. We	C	X	X	X									
	Antañahui	Onagraceae	*Oenothera multicaulis* Ruiz & Pav. (93)	B	F	We	C	X	X	X									
	Yawarjutumi	Orobanchaceae	*Bartsia* aff. *ianaequalis* subsp. *duripilis* (Edwin) Molau (97)	B	G	Wi	C	X	X	X	X					X	X	X	X
	Rosado jutumya	Orobanchaceae	*Bartsia* aff. *santolinifolia* (Kunth) Benth. (96)	B	S	Wi	C	X	X	X									X
	Chulco	Oxalidaceae	*Oxalis peduncularis* Kunth (100)	V. B. D	F. Ri	Wi. R. We	C. To		X	X									
	Pochgo	Oxalidaceae	*Oxalis rigidicaulis* R. Knuth (101)	M	G	Wi	C	X	X	X									
	Ogoro	Phrymaceae	*Erythranthe glabrata* (Kunth) G. L. Nesom (104)	V. B. D	L	Wi	C	X	X	X	X	X	X	X	X	X	X	X	X
	Airampo	Phytolaccaceae	*Phytolacca bogotensis* Kunth (105)	V	S	Wi	C	X	X	X	X	X	X	X	X	X	X	X	X
	Pacha congona, siempre viva	Piperaceae	*Peperomia scabiosa* Trel (109)	B. D	S. F	Wi. R	C	X	X	X	X	X	X	X	X	X	X	X	X
	Congona	Piperaceae	*Peperomia inaequalifolia* Ruiz & Pav (108)	B	H	Wi	Tr	X	X	X	X	X	X	X	X	X	X	X	X
	Siempre viva	Piperaceae	*Peperomia galioides* Kunth (107)	B	Ri	Wi	C	X	X	X	X	X	X	X	X	X	X	X	X
	Llantén	Plantaginaceae	*Plantago lanceolata* L. (110)	B	S. F	Wi. We	C	X	X	X	X	X	X	X	X	X	X	X	X
	Gara llantén, Shapra llantén	Plantaginaceae	*Plantago major* L. (112)	B	S. F	Wi. We	To	X	X	X	X	X	X	X	X	X	X	X	X
	Llantén	Plantaginaceae	*Plantago limensis* Per. (111)	B	S. F	Wi. We	C	X	X	X	X	X	X	X	X	X	X	X	X
	Wayra ogsha	Poaceae	NN 18	B	G	Wi	C	X	X	X	X	X	X	X	X	X	X	X	X
	Pasamagay macho, pacha mullaca	Polygonaceae	*Muehlenbeckia volcánica* Meisn. (115)	B. F	G. S	Wi	C	X	X	X	X	X	X	X	X	X	X	X	X
	Linhuaycerba	Polypodiaceae	*Niphidium macbridei* Lellinger (117)	B	Ri	Wi	C	X	X	X	X	X	X	X	X	X	X	X	X
	Piccahuay	Proteaceae	*Oreocallis grandiflora* (Lam.) R. Br (118)	B	G	Wi	C	X	X	X	X	X	X	X	X	X	X	X	X
	Muchqui	Rosaceae	*Hesperomeles*	B	S	Wi	C	X	X	X	X	X	X	X	X	X	X	X	X
			*cuneata* Lindl. (122)																
	Cerezo, cereza nativa, guinda	Rosaceeae	*Prunus serotina* Ehrh. (125)	F	F. H	Wi	C. Tr		X	X	X	X	X						
	Guapo shiraca	Rosaceae	*Rubus sparsiflorus* J.F. Macbr (128)	F. D	R. F	R. We	C. To		X	X	X	X							
	Chimú hembra	Rubiaceae	*Galium corymbosum* Ruiz & Pav. (129)	B	P	Wi	C	X	X	X	X	X	X	X	X	X	X	X	X
	Chimu	Rubiaceae	*Galium hypocarpium* (L.) Endl. ex Griseb. (130)	B	G	Wi	C	X	X	X	X	X	X	X	X	X	X	X	X
	Chimbo chimbo, Chimu macho	Rubiaceae	*Galium* sp. 1	B	S	Wi	C	X	X	X	X	X	X	X	X	X	X	X	X
	Chimbo chimbo blanco. Chimu hembra	Rubiaceae	*Galium* sp. 2	B	S	Wi	C	X	X	X	X	X	X	X	X	X	X	X	X
	Pasamagay	Rubiaceae	NN 22	B	S. G	Wi	C	X	X							X	X	X	X
	Pasamagay macho	Rubiaceae	NN 27	B	S	Wi	C	X	X							X	X	X	X
	Chamana	Sapindaceae	*Dodonaea viscosa* Jacq (131)	B	S	Wi	C	X	X	X	X	X	X	X	X	X	X	X	X
	Capuli hembra	*Solanaceae*	*Jaltomata sinuosa* (Miers) (132) MioneL.	F	H	We	To, E		X		X								
	Capuli macho	Solanaceae	*Physalis peruviana* L. (133)	F	F	We	To, E, Tr, S	X	X	X									
	Jirka papa	Solanaceae	*Solanum* sp. (135)	B	G	Wi	C		X	X									
	Mashua silvestre	Tropaeolaceae	*Tropaeolum* sp. (136)	B	F	We	To	X	X	X	X								
	Ishanca, Yuraj ishanca, ortiga blanca	Urticaceae	*Urtica urens* L. (137)	B	F	R. We	C	X	X	X	X	X	X	X	X	X	X	X	X
	Yana ishanca, ortiga negra	Urticaceae	*Urtica* sp. 1	B	F	R. We	C. To	X	X	X	X	X	X	X	X	X	X	X	X
	Raíz valeriana, Valeriana	Valerianaceae	*Valeriana pilosa* Ruiz & Pav. (138)	B	G	R	C	X	X	X	X	X	X	X	X	X	X	X	X
	Jacha cedrón, Cedrón de palo	Verbenaceae	*Aloysia citriodora* Paláu (139)	B	S	Wi	To. Tr	X	X	X	X	X	X	X	X	X	X	X	X
	Yawarjutumi	Verbenaceae	NN 29	B	S. R	Wi. R	C	X	X	X	X	X				X	X	X	X
	Verbena	Verbenaceae	*Verbena litoralis* Kunth (140)	B	R. F	R. We	C. To	X	X	X	X	X	X	X	X	X	X	X	X
	Verbena	Verbenaceae	*Verbena* sp. (141)	B	R. F	R. We	C. To	X	X	X	X	X	X	X	X	X	X	X	X

Numbers in parentheses are Pancorbo-Olivera collection numbers [33] deposited in the herbarium MOL at the Universidad Nacional Agraria La Molina, Lima, Perú

Ecological status (ES): Wi, wild; R, ruderal, We, weedy

Use: F, fruits; V, vegetables; B, beverages; D, desserts and condiments, M,  miscellaneous, R, roots, bulbs, and tubers

Management: C, collection; To, tolerance; E, enhancement; Tr, transplantation; S, sowing and planting

Habitat: G, puna grasslands; S, humid savanna; Ri, riparian forest; H, homegarden; F, farm and surroundings; Ro, roads; L, puna lawns

Another group of plants was called “complementary.” There were 41 species in Cani and 92 in Monte Azul, which are plants collected and taken to home for use as condiments or for preparing beverages, but in low quantities (only tender branches and leaves); therefore, they were not quantified (Table 3). Finally, plants that are consumed occasionally only in the place where they are obtained were called “miscellaneous”; these are 10 species in Cani and 16 in Monte Azul.

The use forms of food plants documented in this study are classified as follows:

- *Beverages* Two subcategories were considered: infusions and cold drinks. Infusions are commonly prepared boiled in water and taken to prevent or treat a disease or because they have a nice flavor, such as *muñá* (*Minthostachys mollis*) and *culín* (*Psoralea glandulosa*). Cold drinks are prepared with plants whose parts (mainly stems or flowers) are crushed to obtain juice and then mixed with cold water and sugar. These are the cases of *yana ogoro* (*Calceolaria sp*.) and *uchu uchu* (*Ceratostema peruviana*, Fig. 4g).
- *Vegetables* Three subcategories were considered: plants for soups, stews, and/or salads. These include *yuyo* (*Brassica rapa* subsp. *campestris*, Fig. 4f), *atogo* (*Amaranthus hybridus*), and *quishiu* (*Cyclanthera brachybotrys,* Fig. 4e).
- *Fruits* Fresh fruits highly appreciated by children are *gongapa* (*Vaccinium meridionale*) and *puka satu* (*Thibaudia mellifera*), *capuli hembra* (*Physalis peruviana,  *Fig. 4e) and *capuli macho* (*Jaltomata sinuosa*).
- *Desserts and condiments* The following subcategories were considered: jams (*shira mullaca*, *Rubus* spp.), desserts (*anís*, *Tagetes filifolia*), seasonings (*walmish*, S*enecio condimentarius*, Fig. 4h)  and those used for preparing *ají* sauces (*uchu uchu, C. peruviana*; see Supplementary material 2).
- *Miscellaneous* plants collected and consumed in the field, such as *tuyo* (*Puya nigrescens*), whose stem is sucked to quench the thirst due to its sweetness, and *mascón* (Asteraceae), whose latex is used as chewing gum.

*Management types.* The following types of management practices were identified: (a) gathering (135 species, among them *huiro huiro*, *Senecio canescens* and *gongapa*, *V. meridionale*); (b) in situ tolerance (42 species), including weedy plants associated with crops and tolerated in chakras, such as *yuyo* (*Brassica rapa* subsp. *campestris*) and *bolsa-bolsa* (*Capsella bursa-pastoris*); (c) enhancement (11 species), which are plants on which people carry out practices directed to increase their population density in natural habitats, such as *auja auja* (*Erodium moschatum*); (d) ex situ transplantation (10 species) of complete individuals taken from their natural populations to crop fields or homegardens, such as *rallán* (*Sambucus peruviana*); and (e) ex situ sowing and planting (6 species, for instance *quishiu*, *Cyclanthera brachybotrys*). It should be noted that almost one-third of the plant species recorded were managed in more than one way.

### Food availability

In Monte Azul, several varieties of native potatoes are cultivated through the technique of seed mixture called *huachuy* in Quechua, and the potatoes are harvested between April and May. The *oca, olluco* and *mashua* are harvested between May and June, but under a good storage system, they may be available until December. The consumption of *tocosh* is greater in the months before the harvest of native potatoes. *Tocosh* consists mainly of fermented potatoes, which may be prepared using different techniques, based on putting potatoes in holes where running water passes through and involving several potato varieties. Other tubers and grains were prepared similarly. This is a technique of preservation by which potatoes and other products are available several months after harvest; therefore, these products are particularly important when the availability of potatoes kept in stores decreases. However, *tocosh* is available and consumed throughout the year. In Cani, corn is harvested between June and July and is dried in *huayuncas* (several cobs tied by their bracts and hung in places of the houses out of reach of rodents), especially for protecting seeds but also for consumption. With this and other forms of storage, maize may be available and consumed all year, similar to other crop legumes and grains. Modern varieties of potatoes are sown and harvested throughout the year. Vegetables are available throughout the year, and most fruit trees are harvested during the rainy season. Pumpkin *mazamorra* is consumed between February and March, when it is ripe, and in stews the rest of the year, when it is “green” (Table 3).

Most vegetables and fruits obtained from wild, weedy, and ruderal plants are mainly collected during the rainy season from November to March, but there is another group of species that may be harvested throughout the year, including those used for infusions or condiments (Table 3). Farms and their surroundings provide not only habitats for crops but also for a significant number of plants (55 species), mainly weeds. These environments are followed by humid savanna, which includes 46 species, high-elevation puna grasslands (41 species), riparian forests (15), roads (9 ruderal plant species), and puna lawns (5).

## Discussion

The basic food patterns described for Cani and Monte Azul include feedstuffs, recipes, and preparation techniques based on ancient Peruvian Andean food, such as potato and corn dishes, and techniques such as *machka*, *tocosh*, and *pachamanca* [9, 58]. However, those people who conserve biological and cultural diversity are often among the poorest and most vulnerable [59]. Poverty is a complex issue defined by multiple variables (income, access to services, and nutrition among others); here, we make reference to problems of undernutrition. According to [42], approximately 41% of children under 5 years old in Huánuco suffer chronic malnutrition, and 33% of Peruvian children with this disease live in rural areas. In addition, there is a greater incidence of poverty in people who have a native language as their first language [60]. Paradoxically, approximately 80% of the food consumed in Peru is generated in the Andean region [61]. In addition, it is expected that in the near future, the effects of climate change on some of these local foods could be constrained. This is, for instance, the case of *tocosh* since, as mentioned by [58], the *hualash* varieties of potatoes used to prepare this food could be at risk because of the increase in temperatures projected for the area.

According to [62], in the last century, farmers from the highlands were better nourished than those from the coast because the latter commonly bought refined foods such as flour and sugar, while those from the highlands consumed cereals of high nutritional value. However, Andean food is in constant change, and now, the inclusion of “urban” foods, which most commonly include processed and industrialized food, is more common in the highlands not only in Perú, but also in Argentina or Chile [63–66]. In Cani and Monte Azul, purchased products represent, on average, 40% of the consumed food weight in a year but provide the highest proportion of kcal, carbohydrates and proteins in both communities. However, these values are lower than those reported by [58], who reported that two-thirds of the total calories consumed by Quisqui households were obtained from rice, wheat products, cooking oil, some vegetables, and other products. This contribution is greater than that remembered by peasants 40 to 50 years ago, and it is progressively increasing. The monetary resources needed to buy food are obtained from the sale of crops, and in Cani, this is complemented by temporary jobs in mines, public transportation, the sale of textiles, groceries, desk materials, or prepared meals. Scurrah [67] documented similar food patterns among peasants in communities of Chopcca, Huancavelica, Peru. She considers such a process to be favored by new roads and an increase in the purchasing power of families. A monetization process is also associated with the employment, migration, and commercialization of agricultural products. This process has resulted in an increasing dependence on market products and is related to the fact that Andean settlers believe that these products improve their social status [61] and coincides with similar patterns documented in Peruvian Andean regions like Huánuco (Ambo province) Junín (Junín and Huancayo provinces) and many provinces of Puno, Peru[68]. In other countries of Latin America, as reported by Barreau [65, 66] for Mapuche´s communities in Chilean Andes, the lack of access to the forest or the role of governmental food programs in elementary schools discourages the use of local food like wild plants, among others. That has also been reported by [68] about Peruvian food programs, plus the role of rural public schools that apply an educative approach that is outside the local context and culture [69] even discriminating local foods. Similar patterns have also been documented by Casas et al. [54, 57] among indigenous communities in Mexico.

In the study area, the consumption of native tubers represented 47% of the food weight in Monte Azul (kg/year) and 29% in Cani, which is lower than that reported by [70] for Huánuco (70%). However, on average, the annual per capita consumption of tubers and other locally produced food (squash, corn) was higher than the national average reported by the INEI [71]. However, much of this high consumption coincides with direct observations in the field, possibly because tubers are the main crops providing carbohydrates, and the energy needed by local farmers is greater because they generally perform more intense physical activity than do urban people [57, 72].

Similarly, as reported for other Andean communities [61, 67, 70] and studies in Quisqui [58], food patterns in Cani and Monte Azul are based on tubers and cereals, whose main nutritional contributions are carbohydrates, and have clearly low levels of consumption of animal protein. Guinea pigs are commonly raised by local households, but these and other domestic animals are mainly destined for sale. Moreover, although [70] reported a surplus in the percentages of recommended carbohydrates and proteins in the area, the consumption of calories, iron, and other micronutrients is low. All these findings indicate low nutritional levels in the study area, which is consistent with information reported by INEI [73], even when the two communities studied are part of the first Zone of Agrobiodiversity of Peru [74]. However, this condition in itself is not a guarantee to address the problems of poverty. It is one for attending to the conservation of agrobiodiversity and biocultural diversity, but other factors affect people’s lives. Migration, environmental, and cultural changes also have significant impact.

Despite the facts described, it is known that Andean crops can provide all the nutrients required if they are consumed in a varied diet [63, 75], both in species and varieties. For Huánuco, it has been reported that farm agrobiodiversity contributes to moderately adequate diets among women, at least the minimum needed. These women belong to family units that grow a high richness of crops mainly for self-consumption [76]. In Monte Azul, the varieties of native potatoes are consumed mixed, and this is considered a strength of the traditional food system by Scurrah [67] because of the combination and complementarity of nutrients these crops have. However, as in the communities from Chopcca studied by Scurrah [67], few farmers are managers of most varieties of different crops. This pattern is related to environmental, human cultural, and socioeconomic factors [6, 77].

Although there is an increasing introduction of processed foods into rural Andean communities, our observations coincide with those reported by Tapia and Fries [63], who considered that regional Peruvian meals are still based on native crops and those “andinized” (crops introduced but successfully adapted and adopted, such as broad beans, garlic and onion). This is because these crops have adopted and continued their domestication process, adapting them to the particular socioecological context, and have a traditional meaning that is to a greater extent related to the feeling and life of the Andean population [63]. Consequently, the persistence of consumption of Andean crops represents a form of food sovereignty, understood as people’s right to nutritious and culturally appropriate food [65, 66, 78, 79] and their right to decide about their own food and production system [80].

Regarding wild, weedy, and ruderal plants, although their contribution in terms of weight and calories to the diet is relatively low (14.4% in Cani, 9.6% in Monte Azul of the total food in kg consumed per year), wild vegetables provide more iron than those grown and purchased. Fruits and greens that children collect from the field are rich in iron, carotenes, and micronutrients [63], and collection is not an uncommon practice among farmers in uncultivated lands in Quisqui [58]. However, these elements are under discrimination, considered “food of the poor” [59], which derives from the *layu pita* complex or the contempt of the man who feeds on what the field produced without his intervention, which arose during the colonial period [9].

These plants are also potential resources for use during extreme events, such as droughts, when rainfed crops are seriously affected, or when other foods are scarce [81]. In Ecuador, peasants name these as “famine food” as documented in Cotacachi, where people eat these in times of loss of crops, scarcity, and famine [82]. Similarly, the practices of transplanting, seed sowing, and planting vegetative propagules increase the diversity of farms and orchards, increasing the resilience of agricultural systems and household economies in the face of extreme events; moreover, these practices constitute a genetic reservoir in the case of endangered wild populations [83, 84]. These practices and the high proportion of weedy plants consumed reflect the importance of anthropogenic ecosystems for obtaining edible plants, which has also been reported by other authors from Perú, Argentina, Chile, and México [15, 28, 52, 65, 85–92].

The contribution of micronutrients to vegetables from homegardens appears not to be high; however, homegardens are places where peasants obtain large amounts of some species important for their local cuisine, but something that has been rarely documented in Andes [66]. The consumption of high number of vegetables in homegardens is a common pattern in the Neotropics*,* as shown recently in the review by [93]. In the district of Quisqui, there has been reported by [94] a total of 146 species managed in homegardens, which includes species from the 29 recorded in this study, such as *Amaranthus hybridus, Tagetes elliptica, Cyclanthera brachybotrys*, and *Passiflora tripartita* var. *mollisima.* Moreover*,* our study also confirms the importance of homegardens contributing to greens for the local diet. In Cani, farmers maintain more greens than in Monte Azul (61.5 and 44.4%, respectively), which can be explained by the lower elevation of Cani (2800 m), that allows the growth of a wider number of species there, as studied in other regions of the tropics [93].

It is important to note that the aim of our study was not to report on statistically representative samples; for that purpose, other methodological tools (e.g., surveys and random sampling) would be needed. We conducted semistructured interviews and snowball sampling to analyze in-depth the details of what is known and managed by households and how these are integrated into the local food system. For such an approach, the sample size (24 households) is adequate. Often, the results and experiences from in-depth studies can be used to design research instruments (e.g., surveys) that can be used more quickly with a larger number of respondents. Our study can be considered as a first step of this direction, for the study area and other communities in the region.

The use of different environments, vertical movement upward and downward in the basin for cultivating different species and varieties, and gathering and grazing of different resources, combined with the purchase of food, is a pattern consistent with that described by Murra [17] and Earls [16]. It is the expression of the Andean strategies of resources and landscape management at different altitudinal levels [33] which helps to face risks and compensate for the uncertain availability of particular resources from year to year in a region that is ecologically and climatically unstable [18]. This is a very close pattern to that documented in other regions, such as Mesoamerica [18, 95, 96], where the “strategy of multiple use of appropriation of nature” proposed by Toledo [97, 98] refers to the fact that the indigenous peoples of the world maximize diversity as a strategy that favors the number of options to guarantee their subsistence and minimize risks.

Remarkably, after approximately 10,000 years of agriculture and domestication, traditional groups of the Andean region continue to manage and consume numerous wild and weedy plant species. This reflects that the practice is an important complementary action to cover food needs, especially during periods of scarcity but also to complement nutrients in the diet. All these aspects reveal that food security and sovereignty should be based on the consideration of all these local elements.

## Conclusions

The diversity of food consumed in the study area, as well as the documented ways of obtaining it, reflects a certain level of self-sufficiency among families in their ability to cover basic nutritional requirements while contributing to the conservation of natural resources. These are features of general patterns of use and management of resources and ecosystems, which are typical of the Andean region and traditional cultural groups. Recognizing these factors is the basis for designing strategies for biocultural diversity conservation and food sovereignty in the communities studied and in the rural Andean context in general. Wild and weedy plants are key components of current and historical diets, shaping local cuisine, and therefore deserve special consideration in research and policies for the conservation of biocultural memory.

It is relevant to promote information about the nutritional advantages of consuming local foods in terms of quantities and combinations, their greater nutritional value than industrialized food, and their importance in nutrition programs. Documenting their habitats provides valuable knowledge for conserving and valuing them as potential sources of food for the present and future.

### Supplementary Information

Supplementary Material 1

Supplementary Material 2

## Data Availability

All data obtained in the field are available in this publication. Nutritional data used for evaluations are available in the references provided.

## Abbreviations

BR: Breeding of domestic animals
CHO: Carbohydrates
CENAN: Centro Nacional de Alimentación, Nutrición y Vida Saludable, Peru
CENAPRED: Centro Nacional de Prevención y Reducción de Riesgo de Desastres, Peru
CIZA: Centro de Investigaciones en Zonas Áridas
CR: Crops and derivates
DGAPA: Dirección General de Asuntos del Personal Académico
DGAPA: Dirección General de Asuntos del Personal Académico, UNAM, Mexico
GA: Gathering
HU: Hunting
IDMA: Instituto de Desarrollo y Medio Ambiente, Peru
INEI: Instituto Nacional de Estadística e Informática, Peru
PMA: Programa Mundial de Alimentos (World Food Program)
PNIA: Programa Nacional de Innovación Agraria, Peru
PRO: Proteins
PU: Purchased products
TEK: Traditional ecological knowledge
UPMSI: Unidad de Promoción del Mercado de los Servicios de Innovación
UNALM: Universidad Nacional Agraria La Molina, Peru
UNAM: Universidad Nacional Autónoma de México, Mexico
VIAFFNN: Vulnerabilidad a la inseguridad alimentaria ante la recurrencia de fenómenos de origen natural
