# Supplementary material for: Gathering, agriculture, and exchange: an ethnoecological approach to the study of food patterns and feedstuff sources in communities of the Central Andes, Peru
Source: J Ethnobiol Ethnomed. 2024 Jul 24;20:69. doi: 10.1186/s13002-024-00705-9 (PMC11271047; doi:10.1186/s13002-024-00705-9)
Supplement: Supplementary file 1 — Supplementary Material 1 [file 13002_2024_705_MOESM1_ESM.docx]

**Supplementary material 1. The fermented food called “tocosh”**

| Food product obtained from the fermentation of certain native potato varieties in natural or built wells near to wetlands and streams. The varieties used for *tocosh* are those which have big amounts of starch, being *hualash* and *puka shiri* the most commonly used in the study area.  The wells are prepared checking they have an entrance and a way out for water, many straw is put in the bottom, and the potato is put above it. Finally, potato is covered with stones which prevent the potatoes to move. Once the necessary time for fermentation has passed (4 months to 2 or 3 years), the stones are removed to take the *tocosh* potatoes out of the well. It can be taken out only the amount needed that day, or they can be extracted bigger amounts, which then are dry to preserve them.  The *tocosh* potatoes, fresh or dry, are peered and parboiled with cinnamon and clove. The starch gives to this dish a thick consistency, that is why it is called *tocosh mazamorra*. Sugar or salt can be added. Some variations consist on substituting water by milk or whey, or adding other ingredients like *quinoa* and *apple*. It is consumed in average 5 times a week in Monte Azul and 3 times a week in Cani. In Monte Azul, it provides 11% of the kg consumed on a year, and 7.6% of the carbohydrates.  This fermentation process was also reported for other foods, for instance, corn, pumpkin, *mashua, arracacha* and *oca*. The last one is called *kaya*. |
| --- |
