# Supplementary material for: Gathering, agriculture, and exchange: an ethnoecological approach to the study of food patterns and feedstuff sources in communities of the Central Andes, Peru
Source: J Ethnobiol Ethnomed. 2024 Jul 24;20:69. doi: 10.1186/s13002-024-00705-9 (PMC11271047; doi:10.1186/s13002-024-00705-9)
Supplement: Supplementary file 2 — Supplementary Material 2 [file 13002_2024_705_MOESM2_ESM.docx]

**Supplementary material 2. Main Traditional dishes in Cani and Monte Azul**

| Those that are part of the daily life food:   1. Stews “*segundos”*. Different vegetables, cropped or wild, prepared with oil, garlic, onion and potatoes. Meat can be added, and it is served with rice. 2. Green soup. Water boiled with potato, *olluco*, noodles, salt, and, sometimes, charqui. Minced vegetables are added when serving. Milk, cheese and eggs can also be added. 3. Picante. Stew prepared with chicken or guinea pig, Welsh onion and peanuts or pumpkin seed toasted and milled. 4. Starch “*almidón*”. Starch rich potato varieties are peeled and grated. The starch is squeezed and small spheres are formed with the pulp, which are then parboiled with cinnamon and clove. The starch is added, turning the preparation into a thick *mazamorra*. It is eaten with sugar, and children appreciate it. 5. Casqui. Soup made of *oca*. 6. Pumpkin *mazamorra*. Cook the ripe pumpkin with water and corn flour. It is mixed with sugar and toasted seeds. 7. *Machka*. Dry and milled wheat consumed combined with infusions or water. 8. *Ají*. Spicy sauce made with pepper (*Capsicum* spp.) milled in *batán* (traditional tool for milling) along with other ingredients (onion, garlic, tomato, lemon, cancha, among others) prepared to be served with soups and stews.   Other dishes are characteristic of festivities or special days for each family (birthdays, baptisms):   1. *Pachamanca*. Special dish cooked with different meats (usually beef or pig) seasoned with *chincho* (*Tagetes elliptica*), *walmish* (*Senecio condimentarium*), *huacatay* (*Tagetes* sp.) and other plants, by putting them into a stone oven pre-heated, along with potatoes, sweet potatoes, broad beans and others. 2. *Locro*. Stew made of hen, chicken or other meats, cooked with garlic, onion and potato. Very juicy. 3. Peach dessert. Peaches parboiled with cinnamon, clove, *palo cedron* (*Aloysia citriodora*) and *anis* (*Tagetes filifolia*). Apple and papaya can be added. |
| --- |
